# Supplementary material for: Silver Nanoparticle Targets Fabricated Using Chemical Vapor Deposition Method for Differentiation of Bacteria Based on Lipidomic Profiles in Laser Desorption/Ionization Mass Spectrometry
Source: Antibiotics (Basel). 2023 May 8;12(5):874. doi: 10.3390/antibiotics12050874 (PMC10215128; doi:10.3390/antibiotics12050874)
Supplement: Supplementary file 1 [file antibiotics-12-00874-s001.zip › antibiotics-2369855-supplementary.pdf]

Supplementary materials

Silver nanoparticles targets fabricated using chemical vapor deposition method for differentiation of bacteria based on lipidomic profiles in laser desorption/ionization mass spectrometry

Ewelina Maślak[1], Adrian Arendowski[1], Michał Złoch[1,2]*, Justyna Walczak-Skierska[1], Aleksandra Radtke[3,4], Piotr Piszczek[3,4] and Paweł Pomastowski[1]

[1]Centre for Modern Interdisciplinary Technologies, Nicolaus Copernicus University in Toruń, Wileńska 4 Str., 87-100 Toruń, Poland
[2]Chair of Environmental Chemistry and Bioanalytics, Faculty of Chemistry, Nicolaus aCopernicus University in Toruń, Gagarina 7 Str., 87-100 Toruń, Poland
[3]Department of Inorganic and Coordination Chemistry, Faculty of Chemistry, Nicolaus Copernicus University in Toruń, Gagarina 7 Str., 87-100 Toruń, Poland
[4] Nano-implant Ltd., Gagarina 5/102, Toruń, Poland, 87-100
*Correspondence: michal.zloch@umk.pl; Tel.: +48 56 665 60 60

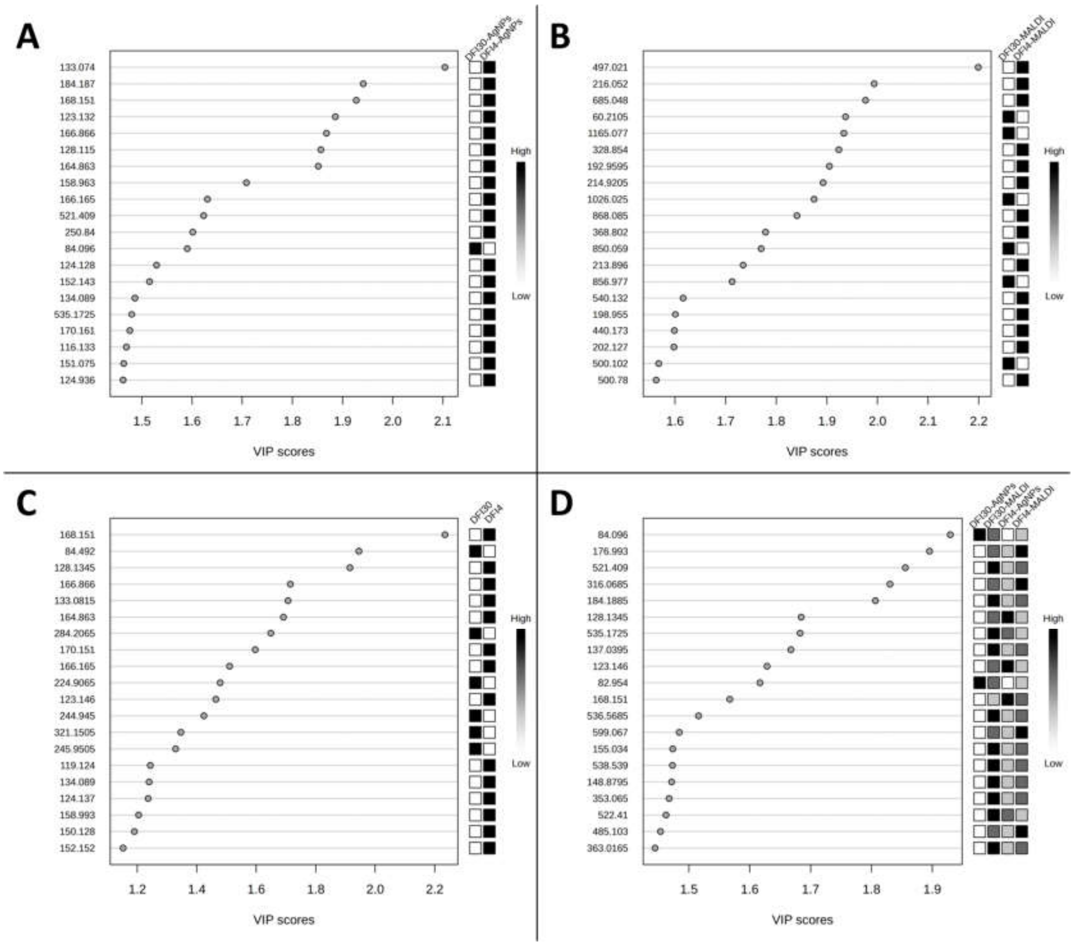

Figure S1. PLS-DA VIP scores for MS data from SALDI/NALDI (A), MALDI (B) experiments and with the use of two groups (C) and four groups (D) for all data.

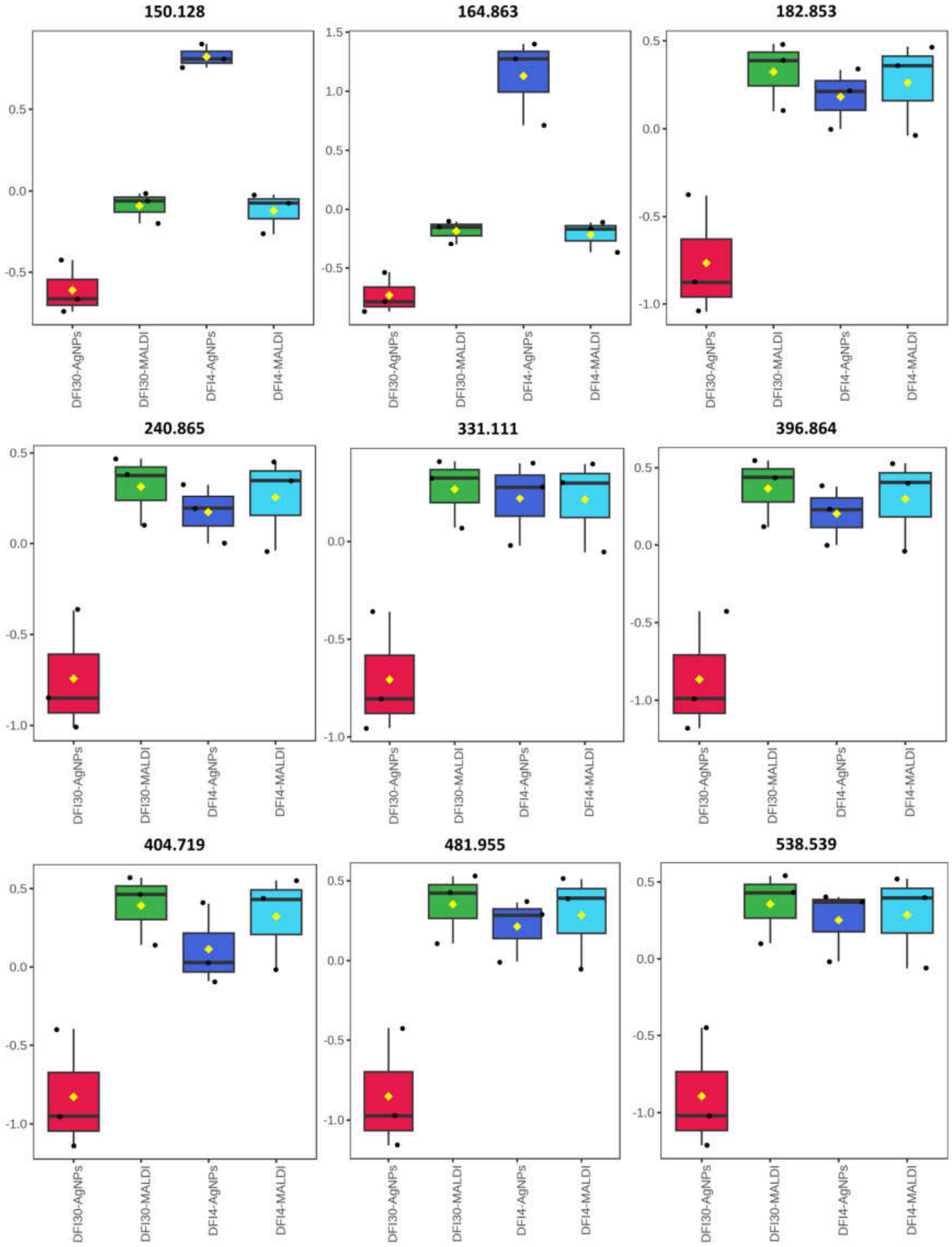

Figure S2. ANOVA box plots for the nine most discriminating *m*/*z* values obtained for DFI4 and DFI30 bacterial lipids samples with use SALDI and MALDI approach.

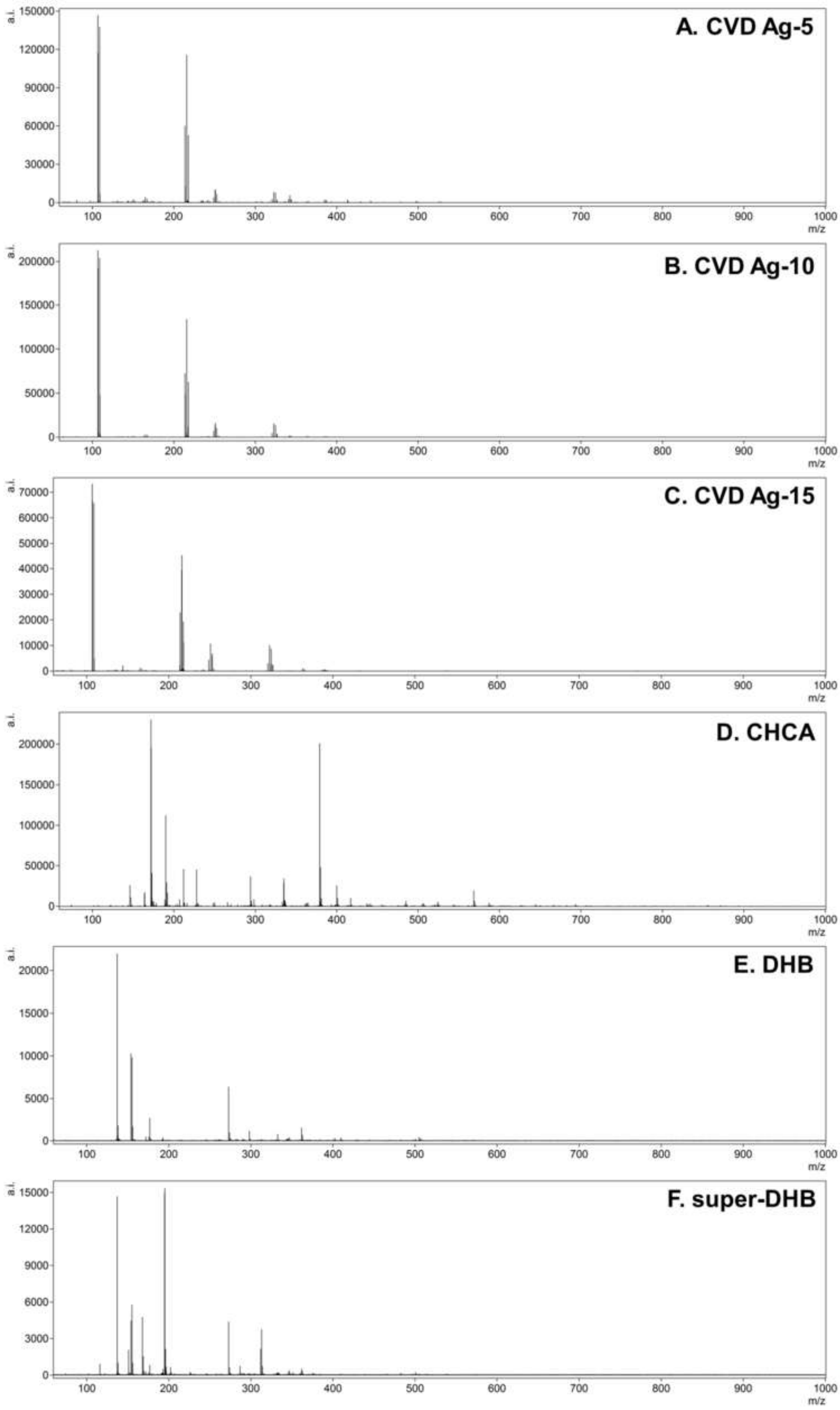

Figure S3. Overview of blank MS spectra performed for SALDI targets with CVD-applied silver nanostructures (A-C) and MALDI matrices (D-F).
